# The role of long non-coding RNAs in cardiac development and disease

**DOI:** 10.3934/genet.2018.2.124

**Published:** 2018-03-26

**Authors:** Carlos García-Padilla, Amelia Aránega, Diego Franco

**Affiliations:** Cardiovascular Development Group, Department of Experimental Biology, University of Jaén, Jaén, Spain

**Keywords:** non coding RNAs, cardiac development, microRNAs, lcnRNAs

## Abstract

Cells display a set of RNA molecules at one time point, reflecting thus the cellular transcriptional steady state, configuring therefore its transcriptome. It is basically composed of two different classes of RNA molecules; protein-coding RNAs (cRNAs) and protein non-coding RNAs (ncRNAs). Sequencing of the human genome and subsequently the ENCODE project identified that more than 80% of the genome is transcribed in some type of RNA. Importantly, only 3% of these transcripts correspond to protein-coding RNAs, pointing that ncRNAs are as important or even more as cRNAs. ncRNAs have pivotal roles in development, differentiation and disease. Non-coding RNAs can be classified into two distinct classes according to their length; i.e., small (<200 nt) and long (>200 nt) noncoding RNAs. The structure, biogenesis and functional roles of small non-coding RNA have been widely studied, particularly for microRNAs (miRNAs). In contrast to microRNAs, our current understanding of long non-coding RNAs (lncRNAs) is limited. In this manuscript, we provide state-of-the art review of the functional roles of long non-coding RNAs during cardiac development as well as an overview of the emerging role of these ncRNAs in distinct cardiac diseases.

## 1. Introduction

**Figure 1.**
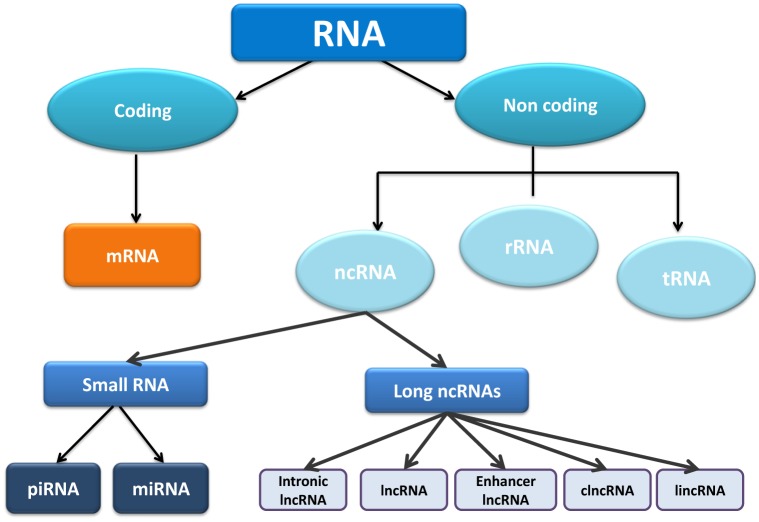
Schematic representation of the distinct classes of RNA molecules.

The cell transcriptome can be defined as the set of RNA molecules present on it at one time point, reflecting thus the cellular transcriptional steady state. It is basically composed of two different classes of RNA molecules; protein-coding RNAs (cRNAs) and non-coding RNAs (ncRNAs) (Figure 1). For decades, scientists focused their attention on coding RNAs, while the non-coding RNAs were defined as the “dark matter” of the genome and were not considered important until recently. An exception to the rule was represented by tRNAs and rRNAs, which were widely studied given their prominent role in protein translation. Sequencing of the human genome and subsequently the ENCODE project identified that more than 80% of the genome is transcribed in some type of RNA. Importantly, only 3% of these transcripts correspond to coding RNAs, pointing that ncRNAs are as important or even more as cRNAs [1],[2]. Currently, it has been demonstrated that non-coding RNAs can perform multiple and important cellular functions acting thus as pivotal regulatory elements in development, differentiation and disease [3],[4].

NcRNAs can be classified into two distinct classes according to their length: (1) Small noncoding RNAs, i.e., smaller than 200 nucleotides; including therein microRNAs, small nucleolar RNAs, piRNAs as well as transfer RNAs, and (2) Long non-coding RNAs, i.e., longer than 200 nucleotides [3], including a extensively variety of types as detailed below (Figure 1). The structure, biogenesis and functional roles of small non-coding RNA have been widely studied, particularly for microRNAs (miRNAs). MiRNAs have an average size of 20–22 nucleotides and act by binding to a target mRNA inducing thereafter degradation and/or inhibition of its translation, and thus negatively regulating gene expression. The functional role of miRNAs have been studied and demonstrated in multiple organisms and within multiple cellular contexts (see for a review; [5],[6]).

In contrast to microRNAs, our current understanding of long non-coding RNAs (lncRNAs) is limited. The development of new massive sequencing techniques has led to the discovery and annotation of a large number of long non-coding RNAs. GENCODE annotation initially estimated the existence of 9640 lncRNA genes in the human genome [7] while recently the NONCODE database has increased this number up to 96,308 lncRNA genes [8]. Such estimates indicate that the number of lncRNAs is twice that coding genes, supporting an important role of these lcnRNA transcripts in multiple biological contexts. Recently, lncRNAs have emerged as major players in regulating gene expression, both at transcriptional and post-transcriptional level and they have been implicated in development, stem cell differentiation, cellular homeostasis and disease [9]–[11].

## 2. Structure and cellular localization of lncrnas

Long non-coding RNAs display essentially no potential to code for proteins, although structurally are similar to mRNAs. They are transcribed using the same pathways; i.e., RNA polymerase II, have typical histone modifications, 5′ terminal cap and 3′ terminal poly(A) tails. LncRNAs are constituted by exons and intronsand are often spliced. Curiously, a minority of non-polyadenylated lncRNAs is transcribed from RNA polymerase III [12]. Unlike mRNAs, lncRNAs have lower number of exons (42% of lncRNA transcripts is composed by two exons compared with 6% protein-coding transcritps) are less conserved between species and on average slightly shorter. Interestingly, lncRNA promoters are more conserved than their exons and in fact almost as conserved as protein-coding gene promoters. Conversely, introns from lncRNAs are longer that those from protein-coding transcripts and are normally flanked by canonical splice sites (GT/AG), showing no differences in splicing signals as compared to protein-coding transcripts [7]. Although the vast majority of lncRNAs are located in the nuclear genome, lcnRNAs are also reported within the mitochondrial DNA. Mitochondrial encoded lncRNAs are transcribed and processed by mitochondrial transcriptional machinery but regulated by nuclear-encoded proteins [13].

Importantly, although lncRNAs they are referred as “non-coding”, several lncRNAs contains short ORFs, can be engaged by ribosomes and thus can generate oligopeptides. Until recently, examples of lncRNAs that could generate small peptides were limited to sporadic cases. However recently, it is becoming increasingly acknowledged that a significant fraction of currently annotated lncRNAs is predicted to be capable of generating short peptides [14],[15]. Among these long non-coding RNAs coding for small peptides, there are several examples, such as *Toddler* and *Dworf*, which are involved in mesoderm development [14]–[16].

LncRNAs display low expression levels yet with increased tissue and time specificity as compared to the protein-coding genes [17]. Such specificity suggests an important role of these transcripts in tightly defined cellular events as supported by several reports [18],[19]. At the cellular level, lncRNAs can be located both in the cytoplasm and nucleus. Cytoplasmic enriched lncRNAs have mainly a role in post-transcriptional regulation whereas nuclearly located lncRNAs predominantly play a role in transcriptional gene regulation. Importantly, lncRNAs are dynamic molecules that can be located in the nucleus but translocate and act in the cytoplasm [20]. An example of such a dynamic behavior is represented by antisense *Uchl1* lncRNA, that partially overlapping *Uchl1* protein-coding gene, moving from the nucleus to the cytoplasm where it bind the 5′ end of *Uchl1* mRNA promoting its translation under stress [1].

Other feature defining lncRNAs is their poor RNA sequence conservation across species, as exemplified by *Braveheart*, a mouse-specific lncRNA involved in early cardiogenesis, as detailed below [21]. However, despite poor conservation between species, the comparison of splice sites suggests that lncRNAs are evolutionarily conserved, showing that the majority of lncRNAs are, as least, as old as mammalian lineage [7].

## 3. Classification of lncRNAs

Classification of long non-coding RNA species differs between authors but at least five distinct groups can be distinguished. Broadly lcnRNAs are classified according to both, their position within the genome and relative location to neighboring genes. (a) lncRNAs transcribed from the same promotor as the adjacent protein coding gene. They are transcribed in both sense and antisense orientation and can be located in the same strand or opposite strand of protein coding gene. The expression of both is correlated and usually these lncRNAs modulates the expression of adjacent protein coding gene. (b) Long intergenic long non-coding RNAs (lincRNAs) are located between two protein-coding genes usually at distance of approximately 10 kb or in genomic desert as stand-alone genes. Since lincRNAs can be transcribed by their own promoter, they are classified as promoter-associated lncRNAs [22]. (c) LncRNAs can arise from intronic regions of protein coding genes, i.e., intronic lncRNA or enhancer regions, i.e., enhancer-associated lncRNA. There is a subclass of intronic lncRNAs, derived from these, known as sno-lncRNAs. Sno-lncRNAs do not have the typical structure observed in the majority of lncRNAs. They are not capped and nor polyadenilated and are flanked by small nucleolar RNAs at both extremes. The enhancer-associated lncRNAs are transcribed from enhancer region and their expression correlates with the expression of active enhancers. Also the expression enhancer lncRNAs correlate with expression of target genes showing a dynamic and specific patterning throughout differentiation and development [23],[24]. (d) Alternative splicing of protein coding genes can generate a circular lncRNA, named circRNAs. These ncRNAs have a great regulatory potential but additional studies are required to fully understand their regulatory mechanisms [25]. For example *Hrcr*, a cardiac enriched circular lncRNA is a protective RNA against distinct molecular mechanisms leading to cardiac hypertrophy [26]. (e) Finally, there is a subclass of lncRNAs harboring microRNAs within their genetic structure, such as H19, which encode miR-675 in its first exon [27].

## 4. Function role of long non-coding RNAs

Long non-coding RNAs are defined as complex non-coding RNA molecules given their particularities affecting their structure as well as their dynamic expression pattern. Such a complexity is reflected in a wide variety of functions. LcnRNAs can act at both transcriptional and post-transcriptional regulation in multiple cellular processes as detailed below.

At the transcriptional level, lncRNAs can modulate the epigenetic landscape of the cell acting as different class of molecules. Currently, four different types of actions have been demonstrated as detail below. Some lncRNAs acting as guide, binding to transcription factors or protein subunits of chromatin remodeling complexes and direct them, as ribonucleoprotein complex, towards their genomic targets, promoting or suppressing gene activity depending on whether the guided complexes are activate (as MLL complex) or repressive complexes (as PRC2 complex). This class of lncRNAs can act in *cis* (i.e., *Xist*) or *trans* (i.e., *HOTAIR*) [28],[29]. For example, *Fendrr*, a cardiac regulatory long non-coding RNA, acts as a guide for PCR2 and Trx/MLL directing them to *Foxf1* and *Pitx2* promoters and setting active and repressive marks [30].

Scaffold lncRNAs can acts as scaffold molecules for different complexes facilitating their assembly and being a functional component of it [31]. For example, *ANRIL*, a long non-coding RNA described as a risk factor for coronary disease acts as platform recruiting and interacting with polycomb complex (PRC1 and PRC2) to the INK4b-ARF-INK4a locus promoting its silencing [32].

Many of lncRNAs are dubbed enhancer lncRNAs since they can act as enhancers of transcription, promoting and maintaining the genomic3D conformation necessary for the transcriptional machinery to get access to promoter regions [33]. Similarly, other long non-coding RNAs can repress the formation of this genomic structures and therefore gene expression. An example of this functional role is *Playrr*, a long non-coding RNA encoded upstream of Pitx2 gene, that represses the expression of this homeodomain transcription factor in asymmetry pathway by interfering with the Pitx2 promoter [34]. The last class of transcriptional regulatory lncRNAs exert their function as decoy molecules competing with transcription factors or chromatin remodeling complexes for their genetic targets. Such interactions avoid that the latter can exert its function and thus indirectly inhibit transcription. *Terra*, telomeric repeat-containing RNA, physically binds to telomerase inverse transcriptase blocking the action of this enzyme and inhibiting telomere elongation [35]. In the cardiovascular context, *Myheart*, a cardiac lncRNA located in the murine myosin heavy chain 7 locus, sequestering BRG1-BAF complex avoiding that this complex can bind to targets [36].

On the other hand, long non-coding RNAs can also regulate gene expression at post-transcriptional level interacting with mRNAs, the translational machinery or other non-coding RNAs such as microRNAs [37]. Some nuclear lncRNAs can participate on pre-mRNA maturation by interacting with pivotal alternative splicing factors. For example, *Malat1* interact with SR (serine/argenine) splicing factors in the nuclear speckle domains and modulates the concentration and distribution of those factors on the nucleus providing proper alternative splicing [38]. As a subclass, these lncRNAs, are dubbed as specific regulators-alternative splicing (sno-lncRNAs). Sno-lncRNAs are located within the nucleolus and in Cajal bodies. This subclass of lncRNAs are associated a several FOX proteins, such as *Fox2* and regulate mRNA alternative splicing in stem cells [39].

Also, long non-coding RNAs can affect mRNA stability by base-pairing with them and altering their half-lives. Depending on efficacy of base pairing, the interaction can promote decoy or mRNA stability. Incomplete base-pairing normally promotes mRNA decoy whereas a full base pairing between both usually promotes mRNA stability and thus protein translation [40]–[43].

Interestingly many lncRNAs are associated with ribosomes and can therefore affect mRNA translation. For example, *LincRNA-21*, an lncRNA co-distributed with ribosomes, represses the translation of different mRNA targets by base pairing at distinct transcript regions, including coding and non-coding regions of mRNAs. This incorrect base pairing generates a complex between *LincRNA-21* target mRNA that interacts with different translation repressors [43]. Also, distinct lncRNAs can interact with the translation machinery by modulating its function. LcnRNA *BC1* interacts, through 3′UTR region, with several translation repressors, inhibiting the assembly of translation initiation complex [44]. On the contrary, *Uchl1*, an antisense lncRNA of *Uchl1* gene, promotes the active polysome generation at the *Uchl1* mRNA enhancing its translation [45]. In the cardiac context, some cytoplasmic lncRNAs act as decoy of mRNA such as *Hcrc* or *Chrr*. Also, lncRNAs can interact with the translation machinery by modulating this. So, BC1 interacts, through 3′ untranslated region, with several represses translation inhibiting the assembly of translation initiation complex [44].

Finally, several lncRNAs can act upon microRNAs interacting with them and modulating post-transcriptional gene expression. *Linc-MD1* can sequester miR-133 and miR-135, enhancing the expression of their target mRNAs in skeletal muscle, such as *Maml1* and *Mef2c*, respectively, among others [46]. In addition, several lncRNAs harboring microRNAs in their genome structure have been described. For example, *H19* contains miR-675 in his first exon. Interestingly, the expression of this microRNA is regulated by *H19* [47].

**Figure 2.**
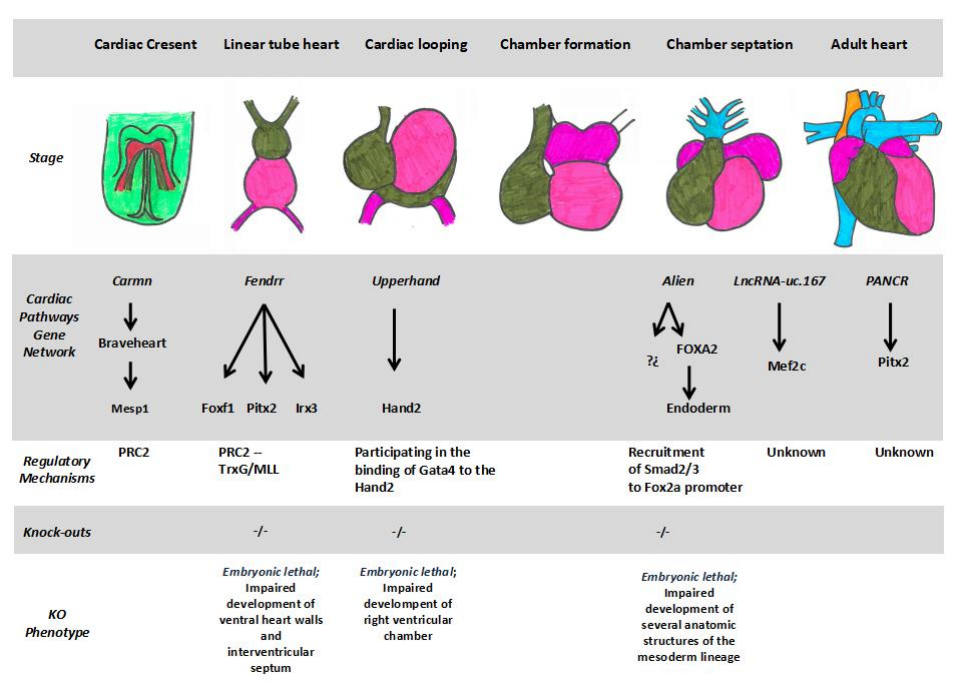
Schematic representation of the distinct stages of heart development from the bilateral sets of precardiac mesoderm (cardiac crescents) to the adult stage, illustrating the distinct long non-coding RNAs described to date and their corresponding molecular signaling pathways. Additional, if knock-out mice are available, the corresponding phenotype is briefly summarized.

## 5. The role of long non-coding RNA in cardiac development

The development of the heart is a complex morphogenetic process and thus highly regulated. The developing heart arises from sets of precursor cells during gastrulation (Figure 2) [48]. These cells progressively converge in the midline of the embryo, forming the early tubular heart [49]. The tubular heart grows and suffers a rightward displacement, a phenomenon dubbed cardiac looping [50], leading to the formation of the different cardiac regions and later a fully functional heart [51]. The molecular mechanisms underlying cardiac morphogenesis include the activation and expression of a several cardiac gene network pathways (CGNPs) evolutionarily conserved. The correct expression of CGNPs is closely regulated in time, specific and spatial patterning by different transcriptional (cardiac-enriched transcription factors, such as Mef2c, Gata4, Nkx2.5 and several members of the T-box family) and post-transcriptional factors (non-coding RNAs such as microRNAs) [52]–[55]. Cardiac transcription factors regulate the transcription of different elements that make up the cardiac transcription pathways forming part of them and regulating transcriptionally the cardiac development [53]. Post-transcriptionally, the role that non-coding RNAs can exert in the regulation of these pathways as well as in other aspects of cardiac development have been described [53]–[55]. Several microRNAs have been post-transcriptionally regulated in the development of the heart interacting with various elements of cardiac signaling pathways [56]. Emerging evidences have pointed out lncRNAs as pivotal players in the heart development regulating transcriptionally and post-transcriptionally distinct cardiac signaling pathways. Transcriptomic analyses have identified a large number of differentially expressed lncRNAs during cardiomyogenic differentiation and proliferation [57],[58]. For example, *HBL1*, a human cardiac-enriched lncRNA, negatively modulates cardiomyocyte differentiation from pluripotent stem cells by interacting with SOX2 and miR-1 [59]. Morever several studies have been performed exploring the functional role of particular cardiac enriched-lncRNAs during cardiogenesis. In particular, to date, seven cardiac lncRNAs have been analyzed in detailed; C*armn, Braveheart, Fendrr, Alien, Upperhand, LncRNA-uc.167* and *Pancr*. We provided here state-of-the art review of the functional role of these lncRNAs in heart development.

### (a). Carmn

Ounzain et al. [60] profiled the lncRNA transcriptome of human cardiac precursor cells and identified a set of 570 lncRNAs differentially expressed during cardiac differentiation. Many of these lcnRNAs were associated with active cardiac enhancers and super enhancers [60]. Super enhancers are associated with increased production of enhancer-associated ncRNAs and with the enrichment of chromatin remodeling complexes and specific histone modifications [61]. Among these lcnRNAs, Ounzain et al. [62] focused their attention on *Carmn*, a super enhancer-associated lncRNA. *Carmn* is located upstream of miR-143 and miR-145, two microRNA involved in cardiovascular development [63],[64]. Although *Carmn*, miR-143 and miR-145 are located within the same genomic locus, they are expressed as independent transcripts. *Carmn* is expressed both in fetal and adult hearts and it is well conserved between mammalian species. *Carmn* directly acts during the earliest steps of cardiac lineage commitment regulating cardiac differentiation from nascent mesoderm by modulating the expression downstream of mesp1-cardiac gene network [62]. Moreover *Carmn* modulates the expression of key factor of pluripotency suggesting a bivalent role both during early differentiation of nascent mesoderm as well as during pluripotency. Interestingly, the expression of *Carmn* in mice modulates, but not in human, the expression of other cardiac-associated lncRNAs, such as *Braveheart*, located downstream of *Carmn*, within the same genomic locus. Mechanistically, *Carmn* acts in *trans* by directly interacting with the polycomb repressive complex 2 (PRC2) through SUZ12, a core protein of this complex, and a EZH2, with mediates methylation of lysine 27 on histone 3, a repressive mark epigenetic that promote the correct cardiac differentiation. The role of this repressive complex in the development of heart has been well reported [65]. Thus *Carmn* exerts an epigenetic function altering the transcriptional landscape by interacting with repressive complex chromatin remodeling. Moreover, *Carmn* is necessary for cardiomyocyte homeostasis, maintaining a differentiated cardiac fate in mature cardiomyocytes [62].

### (b). Braveheart

*Braveheart* is a mouse specific heart-associated lncRNA that plays a pivotal role during cardiac development. *Braveheart* acts as a key regulator in cardiac lineage commitment and it is required for proper cardiac gene expression in mice. *Braveheart* acts upstream of *Mesp1*, and it is nonetheless required for its activation within the same genetic pathway. Depletion of *Braveheart* results in failure of activation of key cardiac factors necessary for correct heart development and cardiomyocyte differentiation. These evidences suggest that *Braveheart* is required to active Mesp1-driven gene expression program and to promote cardiac cell fate from nascent mesoderm. Moreover, *Braveheart* is necessary for fetal and neonatal cardiomyocyte homeostasis and the maintenance of cardiac fate of its [21]. Similarly evidences have also been demonstrated that *Braveheart is* necessary for ESC to acquire cardiac lineage commitment and differentiation into cardiomyocytes [21].

Recently, Xue et al. [66] have determined the secondary structure of *Braveheart* and showing that *Braveheart* adopts a high modular structure with a 5′ AGIL motif that is required for correct mode of action of *Braveheart*. Importantly, this motif is necessary to for cardiovascular lineage commitment and proper ESC differentiation. These authors also demonstrated specific interactions between *Braveheart* and zinc-finger TF CNBP a negative regulator of the cardiac development program repressing the CM differentiation. Thus, it seems that *Braveheart* act as antagonist of CNBP to promote cardiovascular lineage commitment [66].

### (c). Fendrr

*Fendrr* is differentially and transiently expressed at the caudal end of the nascent lateral plate mesoderm, being necessary for the correct development of tissues derived from it, especially the heart and the body walls. *Fendrr* is located 1250 base pairs upstream of *Foxf1* and is co-expressed with this transcription factor. *Foxf1* is of vital importance for the proper differentiation of lateral mesoderm in splanchnic mesoderm and somatic mesoderm [67]. Gene targeting approach has showed that the lack of *Fendrr* carries embryonic lethality at E13.75, characterized by an incorrect heart function, blood accumulated in the right heart chambers and a critical decrease in the thickness of the ventral body walls. The incorrect function of the heart is explained by a hypoplasia of the myocardium that leads to the development impair of ventral heart walls and interventricular septum that are too thin to be able to withstand the blood pressure. Mechanistically, *Fendrr* acts as epigenetic regulatory element by establishing a ratio of repressive and activate marks in the promotors of pivotal transcription factors involved in the mesoderm development, such as *Foxf1*, *Pitx2* and *Irx3.* Such epigenetic regulation is provided through interaction with chromatin remodeling complexes, the histone-modifying Polycomb repressive complex (PRC2) and Trithorax group/MLL protein complex (TrxG/MLL), respectively. The establishment of this ratio determinates the patterns of expression of *Fendrr* target genes in the nascent lateral plate mesoderm and in their descendants of by setting long-term epigenetic marks. *In silico* approaches have showed the existence of 40-nucleotide stretch in the *Fendrr* structure, which is able to interact with *Foxf1* and *Pitx2* promoters, thereby *Fendrr* seem to can be binds directly to those promoters via the formation of a dsDNA:RNA triplex structure. However this interaction needs to be experimentally validated [30],[68].

### (d). Alien

*Alien* is co-expressed in vascular progenitor cells derived from allantoides and lateral plate mesoderm along with genes involved in skeletal muscle development and heart morphogenesis. Gene targeting approaches have showed that the loss of *Alien* results in impaired development of several mesodermal derivatives. Among them defective vascular patterning and cardiac chamber formation is reported [69]. These observations suggest that *Alien* specially acts as pivotal regulator in the cardiovascular development by exerting a function in an early stage of cardiovascular differentiation common to both vascular and cardiac progenitors. The molecular aspects of *Alien* function are unknown to date and thus it will be interesting to study the specific role of this lncRNA in the cardiovascular commitment [69].

On the other hand, *Alien* participates in the endoderm differentiation regulating positively the transcription of *FOXA2*, an important regulator of endoderm development, by facilitating *SMAD2/3* recruitment to the *FOXA2* promoter [70]. Thus Alien acts a versatile RNA molecule during the cardiovascular development.

### (e). Upperhand

A recent study has showed that transcription of a promoter-associated lncRNA located near to *Hand2* is necessary for the expression of this transcription factor and proper heart development. This lncRNA is known as *Upperhand*, is located 150 bases pairs upstream of *Hand2* and shares a bidirectional promoter with this transcription factor. Interestingly, *Upperhand* locus contains a Hand2 associated cardiac enhancer within an intron. *Upperhand* is co-expressed in a temporal and tissue-specific pattern along with *Hand2* during embryonic development. *Upperhand* expression is enriched in the cytoplasm. The function of this lncRNA has been studied by gene targeting approach. *Upperhand* knockout (KO) mice display similar phenotype as*Hand2*KO mice, characterized by a development impairment of right ventricular chamber and embryonic lethality [71]. *Upperhand* KO embryo failed to establish H3K27ac marks in the Hand2-Uph locus and binding of *GATA4* to the *Hand2* cardiac enhancer is also impaired. This molecular interaction is required for the activation of *Hand2* cardiac enhancer and thus for Hand2 transcription [72],[73]. Chromatin immunoprecipitation analyses (ChIP) in *Upperhand* KO embryo have showed that both the loss of the H3K27ac marks and the lack of Gata4 interaction with the *Hand2* cardiac enhancer negatively affects*Hand2* transcription, preventing the RNA polymerase II elongation within the *Hand2* locus. Interestingly, *in vitro* approaches in HL-1 have showed that the mature *Upperhand* transcripts are not required for *Hand2* expression suggesting that is the *Upperhand* transcription, the responsible of *Hand2* expression. Thus, these findings suggest that *Upperhand* transcription is required to the *Hand2* expression by participating in the establishing of the H3K27 marks and in the binding of *Gata4* to the *Hand2* associated cardiac enhancer both necessary to the proper hand2 transcription by the RNA polymerase II [74].

### (f). LncRNA-uc.167

A screening of transcriptome of patients with ventricular septal defect (VSD), has showed the differential expression of a considerable number of lncRNAs [73]. Among them Song et al., [75] focused on *LncRNA-uc.167*, given its prominent expression in VSD patients. *LncRNA-uc.167* is located in the opposite strand of *Mef2c* and is well conversed between species. The expression of both follows an inverse pattern throughout cardiac development and also during the process of P19 cell differentiation into cardiomyocytes. Moreover, the overexpression of *lncRNA-uc.167* results in inhibition of *Mef2c* and absence of differentiation of P19 characterized by a higher level of apoptosis and a slower proliferation rate. The effects of *lncRNA-uc.167* overexpression are partially reduced by *Mef2c* overexpression, suggesting a functional relationship between them. Thus, those observations suggest that *LncRNA-uc.167* can participate in *Mef2c* signaling during heart development [76], yet further experiments are required to fully understand the molecular mechanisms behind *lncRNA-uc.167* and *Mef2c* interaction.

### (g). Pancr

Genome-wide association studies (GWAS) have associated several SNPs (risk variants) in distinct genetic loci with atrial fibrillation, including 4q25 genomic locus [77],[78] where the homeobox transcription factor *Pitx2* is located. *Pitx2* is a pivotal player in embryonic left-right asymmetry pathway and cardiac development [79]. In this context, Gore-Panter et al. [80] exploring the possible relationship between *Pitx2* and these AF risk variants, identified a long intergenetic non-coding RNA adjacent to *Pitx2*, dubbed as *Pancr*. *Pancr* is expressed in the adult left atrium and in lower levels in the adult eye, and shows a coordinate expression with *Pitx2c*, during the differentiation of cardiomyocytes regulating positively the expression of *Pitx2c* mRNA by a yet unknown mechanism. Interestingly, *Pancr* have been reported in human tissues but no orthologues are found in other mammalian species such as mice. Thereby, *Pancr* seems to be a human specific lincRNA [80]. Since, the regulation of *Pitx2c* by *Pancr* it will be interesting to explore the role of this lincRNA in the cardiac development to provide more information of left-right asymmetry pathway.

## 6. Long non-coding RNAs in cardiac diseases

**Figure 3.**
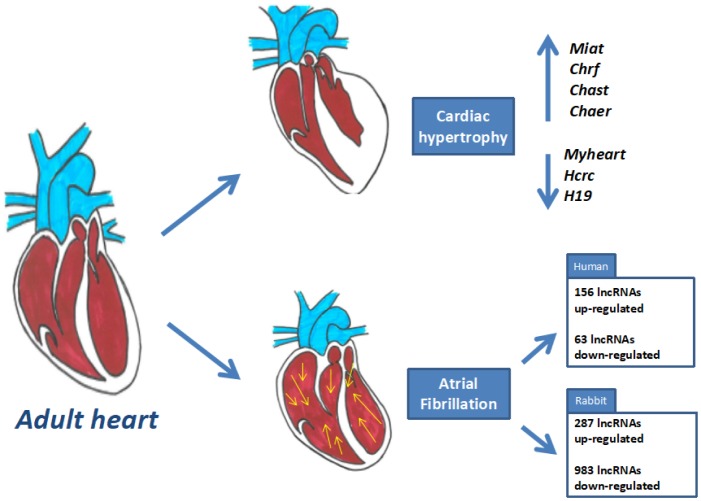
Schematic representation of the distinct cardiac pathophysiological conditions and the distinct long non-coding RNAs described to date.

The role of long non-coding RNAs in disease is recently emerging. Over the last few years, the number of reports that have associated lncRNAs differential expression with some cardiac pathology has considerably increased [4]. Several transcriptomic studies in different species, using deep sequencing, have identified that multiple lncRNAs are deregulated in distinct cardiac pathologies (Figure 3), particularly in acute myocardium infarction [81],[82], heart failure [83]–[85], cardiac fibrosis [86] and atrial fibrillation [87],[88]. These studies have shown that lncRNAs are important players in the maintenance of cellular homeostasis and in disease processes, being their function necessary to correct physiological status.

Several studies have indicated the importance of lncRNAs in cardiac hypertrophy. For example, *Myhear*t, a lncRNA located in the murine myosin heavy chain 7 genomic locus, prevents cardiomyocyte hypertrophy by sequestering BRG1-BAF complex and therefore avoiding that this complex can bind to target genes and induce an hypertrophy response [36]. *Hrcr*, a cardiac circular lncRNA plays a protective role in the cardiac hypertrophy too, acting a decoy lncRNA by sequestering miR-223, considered as pro-hypertrophy factor [26]. Also, H19 and his encoded miR-675, play a protective role in hypertrophy cardiac by modulating cardiac CaMKIIδ expression in the hypertrophy response [27]. Other lncRNAs promote a pro-hypertrophy response in cardiomyocytes, such as *Chast, Chaer, Chrf* or *Miat*. *Chast*, cardiac hypertrophy–associated transcript, is overexpressed by hypertrophy stimuli and its overexpression, independently of pro-hypertrophic factor, is sufficient to activate the hypertrophy response in the cardiomyocytes both *in vivo* and *in vitro* [89]. *Chaer*, cardiac-hypertrophy-associated epigenetic regulator, promotes the hypertrophy by interacting directly with PRC2 and inhibiting the formation of transcriptional silent chromatin complex of pro-hypertrophic factors [90] *Chrf*, cardiac-hypertrophy-response factor, acts a sponge of miR-489, which target is mRNA of pro-hypertrophy factor *Myd88*, thereby promote the hypertrophy response by avoiding that miRNA can degrade *Myd88* transcripts [91]. Finally, *Miat*, myocardial infarction–associated transcript, promote partly cardiac hypertrophy by sponging miR-150, an important miRNA with suppressor effect in the cardiac hypertrophy [92].

Acute myocardial infarction, ultimately leading to heart failure, is frequently associated with a progressive accumulation of fibrotic depositions, i.e., cardiac fibrosis [86]. Differentially expressed lncRNAs have been identified in this fibrotic process. *Wisper*, a cardiac-fibroblast enriched lncRNA conserved between mouse and human, is necessary for the development of cardiac fibrosis. The expression of *Wisper* is required for survival and transdifferentiation of cardiac fibroblasts and maintaining the correct pro-fibrotic gene regulatory network. GapmeRs-mediated attenuation *in vivo* reduces the fibrotic process after myocardium infarct in mice, pointing to *Whisper* as potential therapeutic target [86]. Furthermore, two cardiac-fibroblast enriched lncRNAs modulate the fibrotic process through MMP2 (matrix metalloproteinase-2) modulation, a pro-fibrotic factor expressed in cardiac fibroblasts [93]. *Meg*, a lncRNA conserved in human and mice, promote cardiac fibrosis, upregulating MMP2 expression, while *Gas5* act as negative effector by sponging miR-21, which, in turn, positively regulates the fibrotic process by PTEN/MMP2 pathway [94]. Other examples of cardiac pro-fibrotic enriched sponges lncRNAs is exemplified by *Miat*, acting upon miR-24 and *H19*, acting upon miR-455 [95],[96], respectively.

Interestingly, although the role of lncRNAs in cardiac arrhythmogenesis scarce, two different studies in humans and rabbits, respectively, have identified a subset of differentially expressed lncRNAs in atrial fibrillation, yet their functional roles remains to be established [87],[88].

In addition to those functional roles in adult cardiac pathophysiology, several reports are also emerging in congenital heart diseases. Song et al. [75] have shown the different expression of up to 1500 lncRNAs between normal hearts and hearts from fetuses with ventricular septal defect, a common congenital heart disease. The deregulation of these long non-coding RNAs supports the possible involvement of long non-coding in the development of CHD, however is necessary a greater knowledge about the biology of lnRNAs to understand the role of these in this kind of disease [75].

## 7. Conclusions

Emerging evidences suggest an important role of long non-coding RNA in cardiac development by regulating different cardiac gene network pathways. In this line of thinking, knockdown of distinct cardiac-enriched lncRNAs results in embryonic lethality, reflecting the pivotal role of lncRNAs in this process. However, understanding of the functional roles of long non-coding RNAs is still in its infancy. In the next coming years we will witness further insights into the diversity of regulatory roles of long non-coding RNAs and their interactions with epigenetic, transcriptional and post-transcriptional regulatory layers, not only during cardiovascular development, but also in cardiovascular pathology. In this context, several lncRNAs have been point out as important regulators of cardiac pathological processes such as cardiac hypertrophy. However, scarce information is available in electrophysiological disorders such as Brugada syndrome, LQT and SQT syndromes or arrhythmogenic right ventricular dysplasia (ARVD). Importantly, lncRNAs are identified in peripheral blood samples, opening the possibility of serving as diagnostic biomarkers of different cardiac disease [19]. Therefore, the identification of lncRNAs and the study of their functional roles, both in development and disease, highlight the important of non-coding RNAs as key regulatory elements.
